# A cross-sectional survey on patient safety culture in secondary hospitals of Northeast China

**DOI:** 10.1371/journal.pone.0213055

**Published:** 2019-03-20

**Authors:** Kexin Jiang, Linli Tian, Cunling Yan, Ying Li, Huiying Fang, Sun Peihang, Peng Li, Haonan Jia, Yameng Wang, Zheng Kang, Yu Cui, He Liu, Siqi Zhao, Gamburg Anastasia, Mingli Jiao, Qunhong Wu, Ming Liu

**Affiliations:** 1 Department of Health Policy and Hospital Management, School of Public Health, Harbin Medical University, Nangang District, Harbin, China; 2 Head and Neck Surgery, Department of Otorhinolaryngology, The Second Affiliated Hospital of Harbin Medical University, Harbin, China; 3 Department of Medical, Affiliated Tumor Hospital of Harbin Medical University, Harbin, China; 4 Department of Organization, General Hospital of Benxi Iron and Steel Co, Benxi, China; 5 Office of Academic Affairs, Hebei Medical University, Chang’an District, Shijiazhuang, China; 6 Department of Nursing Psychology and Humanities, Hebei Medical University, Yuhua District, Shijiazhuang, China; 7 Department of Management, I.M. Sechenov First Moscow State Medical University, Moscow, Russian Federation; 8 Chinese Academy of Social Science, Institute of Quantitative & Technical Economics, Dongcheng District, Beijing, China; 9 Department of Social Medicine, School of Public Health, Harbin Medical University, Nangang District, Harbin, China; 10 Otorhinolaryngology, The Second Affiliated Hospital of Harbin Medical University, Harbin, China; Medical University Graz, AUSTRIA

## Abstract

**Objectives:**

This study aims to investigate patient safety culture in secondary hospitals of Heilongjiang, Northeast China, and explore the implications of patient safety culture and practices through the perspectives of various healthcare workers.

**Methods:**

A cross-sectional survey using the Safety Attitude Questionnaire (SAQ) was conducted to ascertain the status of patient safety culture in nine secondary hospitals across the six dimensions of the SAQ. Among the 900 staff members who were invited to participate, 665 completed the questionnaire. Descriptive statistics were used to calculate the general means and standard deviations of the patient safety culture dimensions and other numerical variables, and F-test and a multivariate regression analysis were used to statistically analyze the differences in perceptions of safety culture considering the differences in demographic characteristics. All statistical analyses were performed using SPSS v. 22.0.

**Results:**

The respondents rated job satisfaction as the highest among all six dimensions of the SAQ, followed in order by teamwork climate, working conditions, and stress recognition (the lowest). There were significant differences among the dimensions of patient safety culture and other factors, such as gender, age, job position, and education. Compared with previous studies, teamwork climate and working conditions scores were quite high, while stress recognition score was very low. We also found differences in patient safety culture by demographic characteristics.

**Conclusions:**

The findings revealed the patient safety culture attitudes of healthcare workers in secondary hospitals of Heilongjiang, and provided baseline data for related future research. This evidence may also help government health policymakers and hospital administrators understand related challenges and develop strategies to improve patient safety culture in secondary hospitals of China and perhaps also in other developing countries.

## Introduction

The multiple well-known reports on patient safety published by the Institute of Medicine (IOM) have induced public awareness on the problem[1]. Patient safety is unavoidably influenced by organizational culture[2], and their nexus is usually known as “patient safety culture,” which refers to the organization members’ shared values and beliefs, and the organizational norms related to patient safety[3, 4]. The increase in the awareness of patient safety has generated concern regarding patient safety culture. According to the IOM reports, improving patient safety culture is the biggest challenge in creating a safer health care system; it influences the likelihood of medical errors and personal failure[5]. Moreover, previous studies have focused more on patient safety culture in developed countries than on that in developing countries[6, 7]. In addition, it has been observed more in large general hospitals (tertiary hospitals) than in lower-level hospitals (secondary hospitals). We believe that our research in the Chinese context will help address these gaps.

Recently, the *British Medical Journal* published an eye-catching report, estimating that the deaths caused by medical errors in the United States each year are more than 250,000—making medical errors the third leading cause of death[8]. This finding is undoubtedly troubling as it points to the importance of patient safety culture in medical care and hospital management[9]. The recent administrative change in many hospitals has made the service culture and operating methods patient-centered and has promoted patients’ rights; moreover, doctors and patients are commonly responsible for ensuring patient safety culture[10]. Most previous studies have highlighted the need for medical institutions to develop a strong patient safety culture in order to improve the quality of care and patient safety culture[11, 12].

China, a developing country with a population of nearly 1.38 billion, faces enormous medical and health service tasks, including strengthening patients’ safety and improving the quality of medical care. In November 2016, in Beijing, the Chinese Hospital Association issued “Patient Safety Goals (2017 Version),” a report based on national conditions and actual research to help improve medical care quality[13–15]. It placed great emphasis on the need for development of suitable strategies for improving patient safety culture. Such strategies can be developed based on relevant data, especially data gathered from the Safety Attitude Questionnaire (SAQ)[16, 17], which is undoubtedly the most widely used, among the many measurement tools, and is widely recognized by scholars worldwide for its good reliability and validity.

In China, “secondary hospitals” are regional medical institutions with around 100 to 499 beds, and they generally include county or city hospitals, Chinese medicine hospitals, maternal and child health hospitals, and so on[18]. Secondary hospitals accept referrals from both primary hospitals (fewer than 100 beds) and tertiary hospitals (more than 500 beds), and offer prevention, rehabilitation, and other care services and conduct teaching and research programs. Secondary hospitals in China bear nearly half of all diagnosis and treatment responsibility for the population[19]. Although the importance of secondary hospitals is apparent, there are only few studies that discuss patient safety culture in secondary hospitals; thus, this article considers the various perspectives of doctors, nurses, and other healthcare workers (medical technicians and managers of medical personnel) to better reflect patient safety culture among staff in secondary hospitals of Heilongjiang, Northeast China. We hope that the findings may prove useful to the building of a Hierarchical Diagnosis and Treatment System (an important part of China’s new medical reform plan). As per our knowledge, no study has yet measured patient safety culture in secondary hospitals of China—a gap that this study aims to fill.

## Materials and methods

### Study design and sampling

A cross-sectional survey was conducted in the province of Heilongjiang, China. Considering the sub-regional economic status and population health distribution[20], we investigated areas with good (Daqing), medium (Qiqihar and Mudanjiang), and poor (Jiamusi). Purposive sampling was used to select 15 hospitals. However, only 9 hospitals agreed to participate—3 from Jiamusi, 2 from Mudanjiang, 2 from Qiqihar, and 2 from Daqing. From a sample of 900, we received a total of 665 valid questionnaires, with a response rate of 74%; the respondents included nurses, doctors, other healthcare workers (medical technicians and managers of medical personnel), with a ratio of 250:271:144—close to 5:6:3, a proportion that corresponds with their overall representation in the medical system according to the Heilongjiang Province Health Yearbook[20]. Most respondents had direct contact with patients. Participation was voluntary and anonymous; all answers were confidential and no response was shared with the local management or anyone else. Hospital managers were asked to collect the completed questionnaires a week after distribution, during July–August 2014.

### Measures

The questionnaire drew on the University of Texas SAQ scale for its item pool and content[21]. We used the Chinese version created by Li, which has Cronbach’s α values of 0.91 for the overall scale and from 0.66 to 0.91 for individual scales[22]. There are 30 items in the scale, across the dimensions of teamwork climate (6 items), safety climate (7 items), job satisfaction (5 items), perception of management (5 items), pressure recognition (4 items), and work conditions (3 items). Based on a previous study, the 5-point Likert scale (1 = strongly disagree, 5 = strongly agree) on our SAQ included an additional option of “not applicable”[23]. In addition, some items were added to collect demographic information, including gender, age, education, occupation, and years of work in hospital. Respondents freely chose the most appropriate answer; higher scores indicated more positive attitudes toward patient safety culture.

### Data analysis

Descriptive statistics were used to calculate the general means and standard deviations of the dimensions of patient safety culture and other numerical variables. Analyses were bilateral; the statistical significance level was set to α = 0.05, 95% CI. All SAQ scores were converted to scores out of 100: 1 = 0, 2 = 25, 3 = 50, 4 = 75, 5 = 100; scores for negatively worded items were reversed. The higher the score, the more positive the healthcare worker’s attitude was toward patient safety culture.

An F-test and a multivariate regression analysis were used to statistically analyze the differences in perceptions of patient safety culture of different participants. For each test, p < 0.05 was used as the cut-off value for statistical significance. All statistical analyses were performed using IBM SPSS Statistics v. 21.0.

### Ethical consideration

Ethical approval was granted by the Institutional Review Board of Harbin Medical University before the process of data collection commenced (Project Identification Code: HMUIRB20170016). Approval of participating hospitals was also obtained for conducting the interviews. Participants were anonymous volunteers who had submitted a written informed consent. To ensure anonymity, we destroyed all the completed questionnaires after data entry.

## Results

### Demographic characteristics of respondents

The participation rate was 74%, similar to that observed in related studies (63%–79%)[24, 25], which indicates that responses to the SAQ items can be taken as representative. Most respondents (75.6%) were women. Most respondents belonged to the 25–45 age group; about one-third of the interviewees had been working for 1 to 5 years, and another large group for more than 15 years. More than 80% had obtained an undergraduate education or higher, and most were married. Details are shown below in Table 1.

**Table 1:** Demographic characteristics of respondents.

Demographic Characteristics	Respondents (n = 665)	Frequency (%)
**Sex**		
**Male**	162	24.4
**Female**	503	75.6
**Age group (years)**		
≤25	120	18.0
**25–35**	158	23.8
**35–45**	247	37.1
≥45	140	21.1
**Years of experience**		
≤1	103	15.5
**1–5**	223	33.5
**6–10**	95	14.3
**11–15**	45	6.8
≥15	199	29.9
**Job position**		
**Doctor**	250	37.6
**Nurse**	271	40.8
**Other**	144	21.7
**Educational qualification**		
**Senior High School**	122	18.3
**College**	298	44.8
**Bachelor’s degree and more**	245	36.9
**Marital status**		
**Unmarried**	161	21.6
**Married**	504	72.1

### Perceptions of respondents on patient safety culture dimensions

The overall mean for patient safety culture was 70.22±8.08. Among the individual dimensions, job satisfaction earned the highest score (74.16±11.29), followed in order by teamwork climate (74.05±11.26), working conditions (72.32±13.93), and stress recognition (61.93±18.71). These results are presented below in Table 2.

**Table 2:** Perceptions of respondents on patient safety culture dimensions.

Patient safety culture dimensions (listwise n = 665)	Mean±SD	Number
**Job satisfaction**	74.16±11.29	1
**Teamwork climate**	74.05±11.26	2
**Work conditions**	72.32±13.93	3
**Safety climate**	69.66±11.09	4
**Perception of management**	69.10±12.07	5
**Stress recognition**	61.93±18.71	6
**Total**	70.22±8.08	-

### Respondents’ perceptions of patient safety culture dimensions based on respondents’ characteristics

Table 3 shows significant differences between the dimensions of patient safety culture considering other factors, except number of years of experience, for which there was no correlation. The most obvious impact was that on different job positions: doctors generally scored higher in all dimensions than nurses or other personnel, except for working conditions. In addition, married participants had better patient safety culture perceptions than those who were unmarried. Job positions and education levels had significant relationships with the total SAQ score. A multiple regression analysis with the total SAQ score showed a significant correlation with job position. Details are shown below in Table 3.

**Table 3:** Respondents’ perception of patient safety culture dimensions.

Study subjects	Teamwork climate	Safety climate	Job satisfaction	Perception of management	Stress recognition	Work conditions	SAQ total score
	Mean±SD	Mean±SD	Mean±SD	Mean±SD	Mean±SD	Mean±SD	Mean±SD
**Gender**							
**Male**	73.9±12.2	71.4±12.8	74.3±12.4	71.7±12.6	66.0±18.9	72.1±17.5	429.5±57.1
**Female**	74.1±10.9	69.1±10.4	74.1±10.9	68.3±11.8	60.6±18.5	72.4±12.6	418.6±45.0
***P***_***1***_***-Value***	0.879	0.019*	0.848	0.002**	0.001**	0.816	0.052
***P***_***2***_***-Value***	0.270	0.049*	0.473	0.002**	0.028*	0.729	0.080
**Age**							
≤25	71.3±12.2	68.5±11.3	71.5±12.4	67.8±11.4	60.6±17.1	71.7±13.5	411.5±52.2
**25–35**	74.3±11.3	70.1±11.4	74.4±11.3	67.6±12.8	60.0±21.1	73.1±13.0	419.6±47.9
**35–45**	74.5±10.7	69.7±11.3	74.5±10.7	69.4±12.0	63.9±17.2	72.4±14.0	424±46.6
≥45	75.3±11.0	70.2±10.2	75.5±11.0	71.3±11.6	61.8±19.6	71.7±15.1	425.7±48.1
***P***_***1***_***-Value***	0.027*	0.582	0.031*	0.041*	0.175	0.794	0.337
***P***_***2***_***-Value***	0.037*	0.877	0.035*	0.011*	0.946	0.600	0.078
**Experience (years)**							
≤1	75.6±12.1	69.6±12.0	75.8±12.3	69.7±13.5	57.5±18.2	74.7±14.0	422.8±42.5
**1–5**	72.4±10.8	69.5±11.2	72.5±10.8	68.3±11.5	62.0±20.0	72.4±12.7	417.1±48.7
**6–10**	73.6±11.6	70.1±10.8	73.6±11.65	68.3±13.3	62.5±19.4	73.9±13.0	422.1±44.7
**11–15**	75.6±11.9	70.4±13.0	75.6±11.9	71.6±11.8	61.3±19.1	72.1±15.4	426.7±52.6
≥15	75.0±10.8	69.6±10.2	75.1±10.8	69.5±11.4	64.0±16.7	70.3±15.2	423.3±46.8
***P***_***1***_***-Value***	0.063	0.978	0.064	0.407	0.077	0.076	0.071
***P***_***2***_***-Value***	0.316	0.176	0.273	0.093	0.136	0.001*	0.112
**Job position**							
**Doctor**	75.5±10.9	71.1±11.2	75.7±10.9	69.7±12.9	65.8±19.2	71.4±15.9	429.1±51.4
**Nurse**	73.9±11.7	69.2±10.2	74.0±11.7	67.8±12.2	59.0±18.5	73.5±12.2	417.5±45.1
**Other**	71.6±10.5	68.0±12.3	71.8±10.7	70.61±10.2	60.7±17.0	71.7±13.3	414.4±47.7
***P***_***1***_***-Value***	0.004**	0.021*	0.005**	0.044*	0.000**	0.006**	0.018*
***P***_***2***_***-Value***	0.006*	0.032*	0.008*	0.253	0.064	0.666	0.039*
**Education**							
**High School**	74.1±11.1	69.7±10.5	74.3±11.1	70.0±12.6	56.7±19.0	72.6±14.3	417.4±42.8
**College**	72.9±11.3	69.0±10.3	73.0±11.3	68.6±12.1	62.7±18.2	72.0±13.4	418.2±48.1
**Bachelor’s degree**	75.4±11.2	70.4±12.2	75.5±11.3	69.3±11.8	63.6±18.9	72.5±14.4	426.8±51.0
***P***_***1***_***-Value***	0.035*	0.339	0.30	0.585	0.003**	0.876	0.000*
***P***_***2***_***-Value***	0.232	0.939	0.242	0.950	0.019*	0.959	0.149
**Marital status**							
**Unmarried**	72.4±12.0	67.7±11.4	72.6±12.1	68.0±12.7	61.2±17.9	70.7±15.6	412.6±52.5
**Married**	74.6±11.0	70.3±11.0	74.7±11.0	69.5±11.9	62.2±19.0	72.8±13.3	424.0±48.4
***P***_***1***_***-Value***	0.032*	0.010**	0.042*	0.174	0.577	0.087	0.764
***P***_***2***_***-Value***	0.644	0.016*	0.702	0.975	0.424	0.010*	0.233

**Note**:

*p ≤ 0.05;

** *p ≤* 0.01;

*P*_*1*_ based on ANOVA; *P*_*2*_ based on multiple regression analysis indicated significant differences in the mean scores.

## Discussion

The participating secondary hospitals had high rates of job satisfaction, teamwork climate, and working conditions, while perception of management and stress recognition in these hospitals was found to be low. It was observed that respondents’ perceptions differed based on their gender, work experience, position, education, and marital status. While most respondents in previous studies belonged to specific groups, such as doctors or nurses, our research contributed further by including and comparing not only doctors and nurses but also other healthcare workers such as medical technicians and managers of medical personnel; this helped us capture the perspectives of different staff members within the hospital. According to our previous research on tertiary hospitals[26], the ranking of the scores in Chinese is in the following order: work condition, teamwork climate, job satisfaction, perception of management, stress recognition, and safety climate; all scores are between 70 and 80 and are significantly higher than those observed in secondary hospitals. In addition, we found that, compared with tertiary hospitals, job satisfaction and safety climate were prioritized in secondary hospitals while work condition, perception of management, and stress recognition dimensions were given lesser importance. It is evident that further large-scale research should be conducted on secondary hospitals. The aim of the present study was to investigate the perceptions of patient safety culture among healthcare workers in secondary hospital settings.

Similar to the findings of other studies, the attitude of healthcare respondents to patient safety culture was also generally positive in this study, with a mean score of 70.22. The highest score by domain was for job satisfaction, followed by teamwork climate. This finding also mirrors the findings of some previous studies[27, 28], which have shown that efficiency, quality of work, and teamwork climate among healthcare workers improve with high job satisfaction[29, 30]. On the other hand, some studies have shown that good teamwork may also camouflage the mistakes and safety problems occurring during hospital work in China[31]; therefore, we need to be cautious when deriving implications from the findings. Regarding perception of management, which is second-lowest dimension as per our research, many studies[32–34] have shown that management plays a vital role in the creation of a patient safety culture. Improving patient safety culture at the management level can effectively help in avoiding systemic errors[35]. Unlike the results of previous studies[36], stress recognition in this study was found to be a low dimension. Although several studies on stress recognition have raised doubts about its construct and have considered excluding it from the SAQ[21, 36, 37], the fact remains that stress recognition is very low in Chinese secondary hospitals, and improved education and training on it will improve the performance quality of healthcare staff.

There were significant differences by gender in several dimensions, including safety climate, perception of management, and stress recognition. This finding is consistent with those of other studies[27, 38]. Interestingly, there was no significant difference in job satisfaction by gender, whereas most studies show that women are more likely to be satisfied[39, 40]. The fact that doctors generally scored higher in all dimensions (except working conditions) than nurses and other healthcare workers also does not correspond with the findings of other studies[41–43], especially when compared with that of Norway, in which the result is exactly the opposite. This may be associated with the fact that most patient-safety-culture-related training in secondary hospitals in China are focused on doctors[44, 45], suggesting the need for Chinese secondary hospitals to strengthen patient safety culture training for people other than doctors. At the same time, our study shows that education and stress recognition are positively related—the higher the academic qualification, the more the sensitivity to stress recognition—as has been stated in other works[46]. Finally, compared with unmarried people, married people demonstrated better awareness of patient safety culture, which also corresponds with the findings of other studies[27].

Our study can also help determine how perceptions of patient safety culture differ between hospital workers in China and in other countries[21, 47–49]. With the steady progress in the reform of Chinese public hospitals, many efforts have been made to improve patient safety[50], such as improving hardware facilities and changing management concepts; these are reflected in the high scores on related dimensions. In addition, as reflected by poor stress recognition, fatigue is the norm for workers in large general hospitals in China[51, 52]; however, since patients in secondary hospitals are comparatively few, healthcare workers may feel more relaxed with their institutions being less overburdened. This study of patient safety culture will be a valuable driver of policy and management initiatives to improve patient safety. However, our study is still the first step in patient safety culture research in Chinese secondary hospitals; additional findings are needed to identify more areas that need attention.

### Limitations

Owing to time and resource constraints, we were able to evaluate only 9 secondary hospitals in the Heilongjiang province. Although our findings may not be perfectly representative, they can certainly provide baseline data for further research on patient safety culture in Heilongjiang and other Chinese secondary hospitals.

## Conclusions

The findings of this study revealed the attitudes of healthcare workers toward patient safety culture in secondary hospitals of Heilongjiang. Their job satisfaction, teamwork climate, and working conditions scores were all higher than those observed in previous studies, while their perception of management and stress recognition scores were comparatively lower. This highlights the need to focus more on the weaknesses of Chinese secondary hospitals in order to improve patient safety culture. There were also significant differences between the dimensions of patient safety culture and sex, age, years of experience, position, and marital status. Doctors generally scored higher in all dimensions than nurses or other healthcare workers.

The findings of this study will be useful in identifying specific domain areas that require improvement and developing group-specific remedial plans. The evidence can help government health policymakers and hospital administrators understand the challenges surrounding the issue of patient safety culture in secondary hospitals and develop strategies to improve it. At the same time, the methods employed in this study may provide a reference for future studies and applications in other developing countries.

## Supporting information

S1 Table Demographic characteristics of respondents. (DOCX)

S2 Table Perceptions of respondents on patient safety culture dimensions. (DOCX)

S3 Table Respondents’ perception of patient safety culture dimensions. Note: *p ≤ 0.05; ** p ≤ 0.01; P1 based on ANOVA; P2 based on multiple regression analysis indicated significant differences in the mean scores.
(DOCX)

S1 File Database. (XLSX)
