# Supplementary material for: A cross-sectional survey on patient safety culture in secondary hospitals of Northeast China
Source: PLoS One. 2019 Mar 20;14(3):e0213055. doi: 10.1371/journal.pone.0213055 (PMC6426212; doi:10.1371/journal.pone.0213055)
Supplement: S1 Table — (DOCX) [file pone.0213055.s001.docx]

**Table 1. Demographic characteristics of respondents.**

| **Demographic**  **Characteristics** | **Respondents**  **(n = 665)** | **Frequency**  **(%)** |
| --- | --- | --- |
| **Sex** |  |  |
| **Male** | 162 | 24.4 |
| **Female** | 503 | 75.6 |
| **Age group (years)** |  |  |
| **$\leq25$** | 120 | 18.0 |
| **25–35** | 158 | 23.8 |
| **35–45** | 247 | 37.1 |
| **$\geq45$** | 140 | 21.1 |
| **Years of experience** |  |  |
| **$\leq1$** | 103 | 15.5 |
| **1–5** | 223 | 33.5 |
| **6–10** | 95 | 14.3 |
| **11–15** | 45 | 6.8 |
| **$\geq15$** | 199 | 29.9 |
| **Job position** |  |  |
| **Doctor** | 250 | 37.6 |
| **Nurse** | 271 | 40.8 |
| **Other** | 144 | 21.7 |
| **Educational qualification** |  |  |
| **Senior High School** | 122 | 18.3 |
| **College** | 298 | 44.8 |
| **Bachelor’s degree and more** | 245 | 36.9 |
| **Marital status** |  |  |
| **Unmarried** | 161 | 21.6 |
| **Married** | 504 | 72.1 |

**Table 2.** **Perceptions of respondents on patient safety culture dimensions.**

| **Patient safety culture dimensions**  **(listwise n = 665)** | **Mean±SD** | **Number** |
| --- | --- | --- |
| **Job satisfaction** | 74.16±11.29 | 1 |
| **Teamwork climate** | 74.05±11.26 | 2 |
| **Work conditions** | 72.32±13.93 | 3 |
| **Safety climate** | 69.66±11.09 | 4 |
| **Perception of management** | 69.10±12.07 | 5 |
| **Stress recognition** | 61.93±18.71 | 6 |
| **Total** | 70.22±8.08 | - |

**Table 3.** **Respondents’ perception of patient safety culture dimensions.**

| **Study subjects** | **Teamwork climate** | **Safety climate** | **Job satisfaction** | **Perception of management** | **Stress recognition** | **Work conditions** | **SAQ total score** |
| --- | --- | --- | --- | --- | --- | --- | --- |
|  | **Mean±SD** | **Mean±SD** | **Mean±SD** | **Mean±SD** | **Mean±SD** | **Mean±SD** | **Mean±SD** |
| **Gender** |  |  |  |  |  |  |  |
| **Male** | 73.9±12.2 | 71.4±12.8 | 74.3±12.4 | 71.7±12.6 | 66.0±18.9 | 72.1±17.5 | 429.5±57.1 |
| **Female** | 74.1±10.9 | 69.1±10.4 | 74.1±10.9 | 68.3±11.8 | 60.6±18.5 | 72.4±12.6 | 418.6±45.0 |
| ***P_1_-Value*** | 0.879 | 0.019* | 0.848 | 0.002** | 0.001** | 0.816 | 0.052 |
| ***P_2_-Value*** | 0.270 | 0.049* | 0.473 | 0.002** | 0.028* | 0.729 | 0.080 |
| **Age** |  |  |  |  |  |  |  |
| **$\leq25$** | 71.3±12.2 | 68.5±11.3 | 71.5±12.4 | 67.8±11.4 | 60.6±17.1 | 71.7±13.5 | 411.5±52.2 |
| **25–35** | 74.3±11.3 | 70.1±11.4 | 74.4±11.3 | 67.6±12.8 | 60.0±21.1 | 73.1±13.0 | 419.6±47.9 |
| **35–45** | 74.5±10.7 | 69.7±11.3 | 74.5±10.7 | 69.4±12.0 | 63.9±17.2 | 72.4±14.0 | 424±46.6 |
| **$\geq45$** | 75.3±11.0 | 70.2±10.2 | 75.5±11.0 | 71.3±11.6 | 61.8±19.6 | 71.7±15.1 | 425.7±48.1 |
| ***P_1_-Value*** | 0.027* | 0.582 | 0.031* | 0.041* | 0.175 | 0.794 | 0.337 |
| ***P_2_-Value*** | 0.037* | 0.877 | 0.035* | 0.011* | 0.946 | 0.600 | 0.078 |
| **Experience (years)** |  |  |  |  |  |  |  |
| **$\leq1$** | 75.6±12.1 | 69.6±12.0 | 75.8±12.3 | 69.7±13.5 | 57.5±18.2 | 74.7±14.0 | 422.8±42.5 |
| **1–5** | 72.4±10.8 | 69.5±11.2 | 72.5±10.8 | 68.3±11.5 | 62.0±20.0 | 72.4±12.7 | 417.1±48.7 |
| **6–10** | 73.6±11.6 | 70.1±10.8 | 73.6±11.65 | 68.3±13.3 | 62.5±19.4 | 73.9±13.0 | 422.1±44.7 |
| **11–15** | 75.6±11.9 | 70.4±13.0 | 75.6±11.9 | 71.6±11.8 | 61.3±19.1 | 72.1±15.4 | 426.7±52.6 |
| **$\geq15$** | 75.0±10.8 | 69.6±10.2 | 75.1±10.8 | 69.5±11.4 | 64.0±16.7 | 70.3±15.2 | 423.3±46.8 |
| ***P_1_-Value*** | 0.063 | 0.978 | 0.064 | 0.407 | 0.077 | 0.076 | 0.071 |
| ***P_2_-Value*** | 0.316 | 0.176 | 0.273 | 0.093 | 0.136 | 0.001* | 0.112 |
| **Job position** |  |  |  |  |  |  |  |
| **Doctor** | 75.5±10.9 | 71.1±11.2 | 75.7±10.9 | 69.7±12.9 | 65.8±19.2 | 71.4±15.9 | 429.1±51.4 |
| **Nurse** | 73.9±11.7 | 69.2±10.2 | 74.0±11.7 | 67.8±12.2 | 59.0±18.5 | 73.5±12.2 | 417.5±45.1 |
| **Other** | 71.6±10.5 | 68.0±12.3 | 71.8±10.7 | 70.61±10.2 | 60.7±17.0 | 71.7±13.3 | 414.4±47.7 |
| ***P_1_-Value*** | 0.004** | 0.021* | 0.005** | 0.044* | 0.000** | 0.006** | 0.018* |
| ***P_2_-Value*** | 0.006* | 0.032* | 0.008* | 0.253 | 0.064 | 0.666 | 0.039* |
| **Education** |  |  |  |  |  |  |  |
| **High School** | 74.1±11.1 | 69.7±10.5 | 74.3±11.1 | 70.0±12.6 | 56.7±19.0 | 72.6±14.3 | 417.4±42.8 |
| **College** | 72.9±11.3 | 69.0±10.3 | 73.0±11.3 | 68.6±12.1 | 62.7±18.2 | 72.0±13.4 | 418.2±48.1 |
| **Bachelor’s degree** | 75.4±11.2 | 70.4±12.2 | 75.5±11.3 | 69.3±11.8 | 63.6±18.9 | 72.5±14.4 | 426.8±51.0 |
| ***P_1_-Value*** | 0.035* | 0.339 | 0.30 | 0.585 | 0.003** | 0.876 | 0.000* |
| ***P_2_-Value*** | 0.232 | 0.939 | 0.242 | 0.950 | 0.019* | 0.959 | 0.149 |
| **Marital status** |  |  |  |  |  |  |  |
| **Unmarried** | 72.4±12.0 | 67.7±11.4 | 72.6±12.1 | 68.0±12.7 | 61.2±17.9 | 70.7±15.6 | 412.6±52.5 |
| **Married** | 74.6±11.0 | 70.3±11.0 | 74.7±11.0 | 69.5±11.9 | 62.2±19.0 | 72.8±13.3 | 424.0±48.4 |
| ***P_1_-Value*** | 0.032* | 0.010** | 0.042* | 0.174 | 0.577 | 0.087 | 0.764 |
| ***P_2_-Value*** | 0.644 | 0.016* | 0.702 | 0.975 | 0.424 | 0.010* | 0.233 |

**Note**: **p ≤* 0.05; ** *p ≤* 0.01;

*P_1_* based on ANOVA; *P_2_* based on multiple regression analysis indicated significant differences in the mean scores.
