# Supplementary material for: A cross-sectional survey on patient safety culture in secondary hospitals of Northeast China
Source: PLoS One. 2019 Mar 20;14(3):e0213055. doi: 10.1371/journal.pone.0213055 (PMC6426212; doi:10.1371/journal.pone.0213055)
Supplement: S2 Table — (DOCX) [file pone.0213055.s002.docx]

**Table 2.** **Perceptions of respondents on patient safety culture dimensions.**

| **Patient safety culture dimensions**  **(listwise n = 665)** | **Mean±SD** | **Number** |
| --- | --- | --- |
| **Job satisfaction** | 74.16±11.29 | 1 |
| **Teamwork climate** | 74.05±11.26 | 2 |
| **Work conditions** | 72.32±13.93 | 3 |
| **Safety climate** | 69.66±11.09 | 4 |
| **Perception of management** | 69.10±12.07 | 5 |
| **Stress recognition** | 61.93±18.71 | 6 |
| **Total** | 70.22±8.08 | - |
